# Effect of Mimic Hypoxia on the Proliferation and Expression of *miR-27a*, *miR-9*, *miR-370* and their Target Genes in MOLT-4 and KG1a Cell Lines

**DOI:** 10.31557/APJCP.2021.22.6.1975

**Published:** 2021-06

**Authors:** Behnam Emaogolizadeh Gurd Tapeh, Ali Mohammadi, Mohammad Reza Alivand, Saeed Solali

**Affiliations:** 1 *Department of Hematology, Tabriz University of Medical Sciences, Tabriz, Iran. *; 2 *Hematology and Blood Banking, Tabriz University of Medical Sciences, Tabriz, Iran. *; 3 *University of Odense, Iran. *; 4 *Department of Medical Genetics, Tabriz University of Medical Sciences, Tabriz, Iran. *

**Keywords:** AML, ALL, cancer, drug resistance, mimic hypoxia, miRNAs

## Abstract

**Objective:**

The aim of this study was to investigate the effect of mimic hypoxia on proliferation, the expression of significant miRNAs, and genes involved in drug resistance in *MOLT-4* and *KG1* cell lines.

**Materials and Methods:**

The KG1 and MOLT-4 cell lines were cultured in RPMI 1640 medium supplemented with 20% FBS and 10% FBS respectively. The MTT test was used for determining the optimum dose of CoCl_2_ for KG1 and MOLT-4 cell lines. Western blotting was used for the detection of HIF-1a protein and the confirmation of mimic hypoxia induced by CoCl_2_. For evaluating the effect of mimic hypoxia on proliferation of MOLT-4 and KG1 cell lines, cell counting was done using trypan blue at 24, 48, and 72 hours. Furthermore, the results obtained from cell counting were confirmed with the MTT test. Total RNA was extracted using the RNX Plus solution kit according to the manufacturer’s protocol. The expression of genes and miRNAs was evaluated with real time PCR.

**Results:**

According to this study, mimic hypoxia induced by CoCl_2_ contributes to the overexpression of drug resistance related genes including* MDR1, MRP1, FOXM1*, *BCL-xl* genes, and the suppression of *PUMA* gene compared to the control group. The results also showed that mimic hypoxia condition leads to the up-regulation of miR-9 and down-regulation of miR-27a and miR-370. Additionally, our outcomes demonstrated that mimic hypoxia has an inhibitory effect on the proliferation of MOLT-4 and KG1 cell lines.

**Conclusion:**

Treatment with CoCl2 has an inhibitory effect on the proliferation of MOLT-4 and KG1 cell lines independent from real hypoxia. Additionally, mimic hypoxia has a substantial effect on the expression of genes and miRNAs involved in drug resistance. Finally, we are still far away to discover the exact functional mechanisms of hypoxia on drug resistance but these evaluations can provide new perspectives into this field for the upcoming studies.

## Introduction

Acute lymphoblastic leukemia (ALL) is one of the most important causes of cancer-related deaths in young children and adults (Champlin and Gale, 1989). ALL is the most common form of leukemia (25–30%), and the majority of patients suffering from ALL are children (75–80%). The average age at diagnosis is 13 years, and the peak of incidence is at 2–3 years of age (Hoffbrand et al., 2016). Another form of leukemia is acute myeloid leukemia (AML), which is characterized by an increase in the number of myeloid cells in the bone marrow, the arrest of maturation and differentiation of these cells (Lowenberg et al., 1999). The annual prevalence of AML in children is 2–3 cases per 100,000 children. Additionally, the annual incidence of AML in adults is 15 cases per 100,000 individuals. The peak of the incidence of AML is in the 7th decade of life (Hoffbrand et al., 2016).

miRNAs, as small, non-coding RNAs play an important role in the pathogenesis of various cancers, including leukemia. miRNAs regulate the expression of genes involved in different cellular processes at the post-transcriptional level (Sugita et al., 2014). Recent studies have reported abnormal expression of miRNAs in various cancers. The fundamental role of miRNAs in the sensitivity of tumor cells to chemotherapy agents has also been reported (Fan et al., 2016; Han et al., 2016).

Today, hypoxia has been considered as a significant factor in the growth of tumor cells. The hypoxia-inducible factor-1a (HIF-1a) is one of the most important regulators of cellular response to hypoxia. HIF-1a is a transcription factor that consists of two non-identical alpha and beta subunits. The alpha subunit is stable under hypoxic conditions, but it quickly disappears at normal oxygen pressures through proteasomal degradation. The beta subunit is also expressed continuously (Kaur et al., 2005). Cobalt chloride (CoCl_2_) has the potential ability to block the degradation of HIF-1a. Therefore, it can be used to create mimic hypoxia condition (An et al., 1998).

Prevention of drug resistance and the relapse of treatment are the most important challenges in treating leukemia patients. The bone marrow compartment is heterogeneous and hypoxic. Additionally, inhibition of HIF-1a hydroxylation contributes to the over-activation of this transcription factor (Baharaghdam et al., 2018). Under hypoxic conditions, the expression of hypoxia-responsive miRNAs (HRMs) is altered. Depending on the cancer cell type, some miRNAs act as tumor suppressors, and others act as oncogenic miRNA; therefore, abnormal expression of miRNAs contributes to tumor suppression or cancer cell development. Recent studies have reported that tumor microenvironment is the main factor in the progression of cancer cells and resistance to radiotherapy and chemotherapy agents (Jin et al., 2014).

It is now clear that specific physiological conditions of the tumor microenvironment, such as hypoxia, are extremely important in the development of cancer. An increase in the HIF-1a level contributes to the changes in the expression of genes and miRNAs containing hypoxia responsive elements (HREs) in the promoter. Therefore, hypoxia can play an important role in the resistance of tumor cells to chemotherapy agents through regulating the expression of genes involved in various cancer cell processes (Jin et al., 2014).

Multiple drug resistance (MDR) is one of the most important causes of recurrence and unsuccessful chemotherapy in cancer. Various mechanisms of MDR have been found, including changes in cell checkpoints, deficits in apoptotic mechanisms, repair of damaged cells, alterations in the intracellular iron concentration, and decreasing of drug accumulation (Abedi et al., 2014). The formation of MDR phenotype is mainly due to overexpression of energy-related pumps (ABC-ATP binding cassette transporters) in cancer cells. Among the ABC transporter members, ATP Binding Cassette Subfamily B Member 1 (*MDR1*) and ATP Binding Cassette Subfamily C Member 1 (*MRP1*) genes, located on chromosomes 7 and 6, respectively, have shown the maximum relation with drug resistance in leukemia (Abedi et al., 2014).

In general, hypoxia has a fundamental role in regulating cancer cell resistance to chemotherapy. In this study, to clarify the biological effects of mimic hypoxia on cancer cells, we selected the most significant miRNAs and their target genes that are involved in cancer cell resistance to chemotherapy agents. 

## Materials and Methods

*Cell culture*

The KG1 and MOLT-4 cell lines were purchased from the cell bank of Pasteur Institute (Tehran, Iran). The MOLT-4 cells are derived from the peripheral blood of a 19-years old male with acute lymphoblastic leukemia in relapse. Additionally, The KG1 cells are derived from a bone marrow aspiration obtained from a 59-year-old Caucasian male with erythro leukemia that evolved into acute myelogenous leukemia. Subsequently, the KG1 and MOLT-4 cells were suspended and cultured in RPMI 1640 (Gibco Laboratories, Grand Island, NY) medium supplemented with 20% FBS (Fetal Bovine Serum) and 10% FBS, respectively, followed by incubation under 5% CO_2_ at 37ºC in a humidified atmosphere. 

*MTT and cell proliferation assay*

The induction of HIF-1a is dose-dependent. For determining the appropriate concentration of CoCl_2_, approximately 20000 MOLT-4 cells and 15000 KG1 cells were transferred to specific wells of the 96-well plates. To reach the highest HIF-1a ratio with no significant effect on cell death, the MOLT-4 cells were treated with different concentrations of CoCl_2_ (0, 25, 50, 100, 150, and 200 µM); also, for the KG1 cell line, different concentrations of CoCl_2_ (0, 200, 400, 600, 800, 1000, and 1,200 µM) were employed. The plates containing KG1 and MOLT-4 cells were then wrapped in aluminum foil and incubated until the incubation time. The test was performed in 5 replicates at the times of 24 and 48 hours. After incubation, the wells were fixed for each time with 30 µL of 5-mg/mL MTT solution commonly used in this assay. After four h of incubation at 37°C, 100 µL of DMSO and 25 µL of Sorensen buffer were added. The plates were then placed on the shaker for 20 minutes at 37°C. Finally, the percentage of living cells was determined by the resultant OD at 570 nm. The MTT test was also used for evaluating the effect of mimic hypoxia on the proliferation of MOLT-4 and KG1 cell lines.

*The treatment of cell lines with CoCl2 for the induction of mimic hypoxia*

Totally, 2×10^6^ KG1 and MOLT-4 cells were treated with 400 μM and 75 μM of CoCl_2_ under 5% CO_2_ and 37°C in an incubator with a humidified compartment for 24 and 48 hours.

*Western blot assay*

Cells were washed twice with ice-cold PBS and lysed in 100 μl of RIPA buffer at 4°C for 15 minutes. The lysates were centrifuged at 12000 x g for 15 minutes. The precipitates were removed and the solutions were stored at -80°C. Protein concentration was measured by Bio-Rad Protein Assay. Briefly, cellular protein 60 mg was resolved on SDS-PAGE and transferred to nitrocellulose membranes. Membranes were probed with the Indicated antibodies (Mouse anti-human HIF-1 alpha, Bio-Rad) and signals were detected with the Enhanced chemiluminescence (ECL) kit (Najm Biotech). The westerns were routinely normalized using actin beta.

*RNA extraction and cDNA synthesis*

A portion of the treated cells was transferred into predetermined falcon tubes to extract the total RNA. At first, for homogenization, the KG1 and MOLT-4 cells were centrifuged for 5 min and the supernatant was removed. Subsequently, both of the KG1 and MOLT-4 cells were suspended and incubated at room temperature for 5 min. To extract the total RNA from the medium containing MOLT-4 and KG1 cell lines, the RNX Plus solution kit was employed according to the manufacturer’s protocol. In the next step, the products obtained from the KG1 and MOLT-4 cells were stored at -70°C. The extracted RNA was used to produce single-stranded cDNA using a high-efficiency kit (Bioneer, Alameda, CA, USA). cDNA synthesis was performed in a reaction mixture containing 500 ng of total RNA, 5 μL of Oligo (dT) primer, 0.5 of μL of random hexamer primer, 5 μL of reverse transcriptase, and 10 μL of water. Finally, the samples were slowly mixed and incubated at 37°C for 15 min and 85°C for 5 sec in a thermocycling machine.

*PCR and Real-time PCR*

Conventional PCR testing was performed using a 2x master mix to confirm the synthesized cDNA. Each reaction contained 10 μL of 2x master mix, 0.25 μL forward primer (10 pmol), 0.25 μL of reverse primer (10 pmol), 1 μL of cDNA template, and 8 μL of nuclease-free water in a total volume of 20 μL. The following cycling program was carried out in the thermocycling machine: One cycle at 93°C for 5 min, 40 cycles at 93°C for 30 sec, 40 cycles at 62°C for 40 sec, 40 cycles at 72°C for 30 sec and one final cycle for 4 min at 72°C. For each gene, the specific forward and reverse primers were used. Beta-actin was used as an internal control. A negative control sample containing all of the PCR primers except cDNA was also used. After the PCR reaction, to ensure the properties of the amplified product, electrophoresis was carried out on 2% agarose gel.

The expression level of mRNAs, including P53 upregulated modulator of apoptosis (PUMA), forkhead Box M1 (*FOXM1*), BCL2 Like 1 (*BCL-xl*), MDR1, and *MRP1* genes were quantitatively evaluated by RT-PCR using SYBER green PCR master mix (amplicon 2xmaster mix) and the Corbett system. Additionally, the expression of miR-27a, miR370, and miR-9 was also evaluated by RT-PCR. Each reaction contained 7 μL of 2x SYBR green mix, 0.3 μL of forward primer (10 pmol), 0.3 μL of reverse primer (10 pmol), 1 μL of cDNA template, and 4.5 μL of nuclease-free water in a total volume of 14 μL. The following cycling program was carried out on the qRT-PCR machine of Corbett: One cycle at 95°C for 15 min, 40–45 cycles, including 95°C for 20 sec, 60°C for 35 sec, 72°C for 25 sec and one final cycle for 5 min at 72°C. The expression ratio of the genes and miRNAs were calculated with the use of the 2(^-ΔΔCT^) method, and beta-actin was used as an internal control. To evaluate the quality of the real-time PCR test, the melting curve analysis was performed using Rotor Gene TM 6000 Real Time Rotary Analyzer software. For each gene, a triplicate test was performed. The list of the primers has been presented in Table 1.

*Statistical Analysis*

All the data were presented as the mean±SD and were analyzed using Prism 8.0 software (Graph Pad). The significance of differences from the control values were determined with 2-tailed Student’s t-test or 1-way ANOVA; a P-value of <0.05 was considered significant. 

## Results

*MTT and cell toxicity assay for MOLT-4 and KG1 cells treated with CoCl*_2_

**Figure 1:**
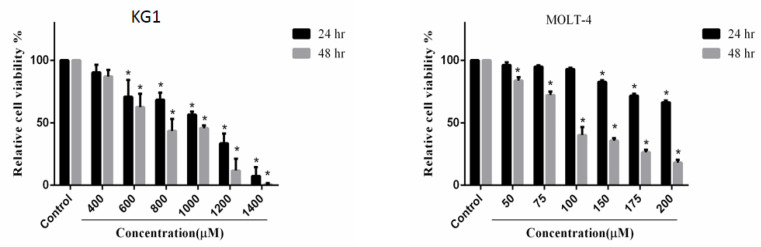
The KG1 and MOLT-4 Cells Treated with Various Concentrations of CoCl_2_ at 24 hours and 48 hours. The KG1 cell lines were treated with 0, 400, 600, 800, 1000, 1200, and 1400 μM concentrations of CoCl_2_ for 24 hours and 48 hours. Based on the results of MTT assay, to create mimic hypoxia with low cytotoxic effect of CoCl_2_, the concentration of 400 μM was selected as the non-cytotoxic dose of CoCl_2_ for 24 hours and 48 hours in KG1 cell lines. Figure 1 also shows the MOLT-4 cells treated with various concentrations of CoCl_2_ at 24 h and 48 h. The MOLT-4 cell lines were treated with 0, 50, 75, 100, 150, 175, and 200 μM concentrations of CoCl_2_ for 24 hours and 48 hours. Based on the results of MTT assay, to create mimic hypoxia with low cytotoxic effect of CoCl_2_, the concentration of 75 μM was selected as the non-cytotoxic dose of CoCl_2_ for 24 hand 48 hours in MOLT-4 cell lines

MTT test for KG1 and MOLT-4 cells demonstrated that the concentrations of 400 μM and 75 μM of cobalt chloride are optimum doses for the induction of mimic hypoxia in these cells, respectively. Based on the results of our study, the cytotoxic effects of cobalt chloride were very low in these concentrations. Additionally, the MTT test indicated that different concentrations of CoCl_2_ have an inhibitory effect on the proliferation of MOLT-4 and KG1 cell lines (Figure 1).

*Western blot assay*

**Figure 2:**
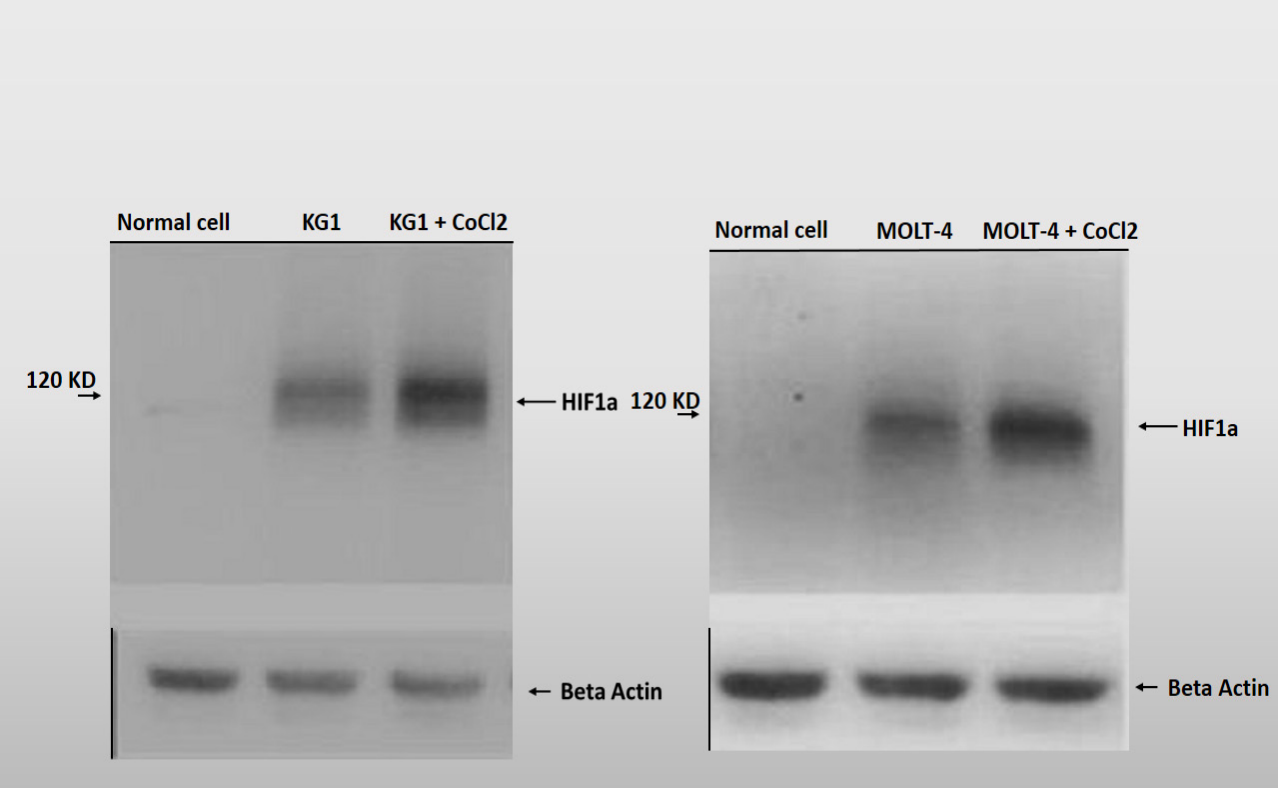
Western Blot Assay to Detect the Expression Levels of HIF-1a in MOLT-4 and KG1 Cell Lines. The KG1 and MOLT-4 cells were treated with 75 and 400 µM of CoCl2 for 48 hours respectively. Consequently, the expression levels of HIF-1a were evaluated with western blot assay

The induction of mimic hypoxia was confirmed through detecting HIF-1a protein by western blotting. Western blot assay demonstrated that CoCl_2_ has induced the mimic hypoxia. After 48 hours, the expression of HIF-1a protein was increased in MOLT-4 and KG1 cell lines. It was important to note that cancer cells are under hypoxic conditions, so even in untreated groups with CoCl_2_, HIF-1a protein is partially expressed in cancer cells. In contrast, the normal cells are not under hypoxia conditions. Accordingly, as Western blot assay showed, the expression of HIF-1a protein was not detectable in normal cells. After 48 hours of treat with CoCl_2_, the expression of HIF-1a increased compared to control and normal cells (Figure 2).

*The effect of mimic hypoxia on the viability of MOLT-4 and KG1 cells*

**Figure 3:**
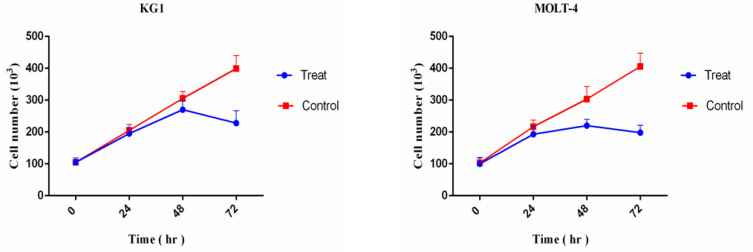
The Effect of Mimic Hypoxia on Proliferation of KG1 and MOLT-4 Cells. The KG1 cells were treated with 400 μM of CoCl2 for 0, 24, 48, and 72 hours. The MOLT-4 cells were also treated with 75 µM of CoCl2 for 0, 24, 48, and 72 hours. The trypan blue at 1:1 ratio was used for cell counting at 0, 24, 48 and 72 hours. According to the results of cell counting, the number of KG1 and MOLT-4 cells are increased for 48 hours under mimic hypoxia condition. Paradoxically, after 48 hours, the number of both cell lines are decreased under mimic hypoxia condition. In addition, under mimic hypoxia condition, the number of KG1 and MOLT-4 cells were lower than the control group at the all of times.*Statistically significant difference in comparison with respective data of MOLT-4 control cells (P<0.05)

After cell counting with trypan blue in 1:1 ratio, cell growth curves for the treated and untreated MOLT-4 and KG1 cells were plotted in Figure 3. According to the curves in Figures 3, under mimic hypoxia condition, the number of MOLT-4 and KG1 cells is increased until 48 hours. Paradoxically, after 48 hours, the number of cells is decreased until 72 hours. In addition, under mimic hypoxia condition, the number of KG1 and MOLT-4 viable cells was lower than the control group at all times. Based on these results, mimic hypoxia contributes to the reduction of the number of viable cells and has an inhibitory effect on cell viability (Figures 3).

*The impact of mimic hypoxia on gene expression*

**Figure 4:**
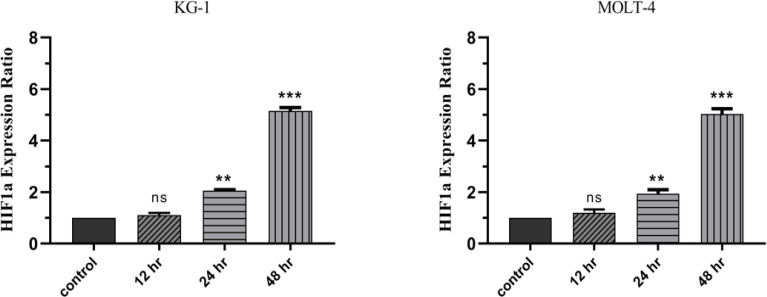
Real-Time PCR Data for HIF-1a Expression in MOLT-4 and KG1 Cell Lines under Mimic Hypoxia Condition (75 and 400 μM of CoCl_2_, respectively). The expression of this gene was evaluated by real-time PCR in MOLT-4 and KG1 cell lines under mimic hypoxia condition. The extraction of RNAs were performed 12 hours, 24 hours, and 48 hours after exposure to CoCl_2_. Data were obtained from the means and standard deviations of three independent experiments.*Statistically significant difference in comparison with respective data of MOLT-4 and KG1control cells (P<0.05).

**Figure 5:**
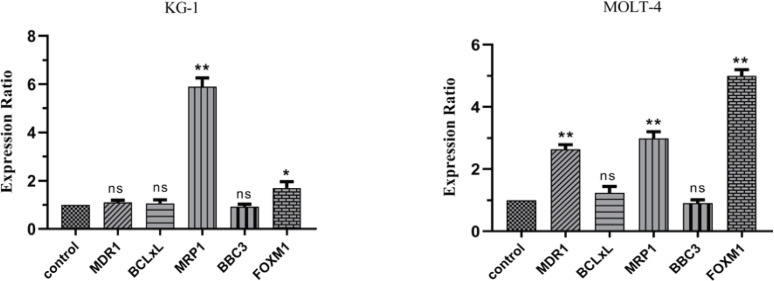
Real-Time PCR Data for MDR1, MRP1, PUMA, FOXM1, BCL-xl Expression in MOLT-4 and KG1 Cell Lines under Mimic Hypoxia Condition. The expression of these genes was evaluated by real-time PCR in MOLT-4 and KG1 cells under mimic hypoxia condition. Extraction of total RNAs were performed 24 hours after exposure to 75 and 400 μM of CoCl2 respectively. Data were obtained from the means and standard deviations of three independent experiments. *Statistically significant difference in comparison with respective data of MOLT-4 control cells (P<0.05).

According to the results from this study, the toxic effects of cobalt chloride occurs after 48 hours. Consequently, fundamental changes occur in cell biology. Accordingly, the best time to evaluate the effect of mimic hypoxia on the expression of genes involved in cellular processes is the time of 48 hours. Additionally, the induction of HIF-1a begins after 24 hours and the cells are completely subjected to mimic hypoxia conditions at the time of 48 hours. The expression of all genes, including *MDR1, MRP1, PUMA, BCL-xl, FOXM1*, and *miRNAs* including *miR-9, miR-370, miR-27a, *compared to the control cells were non-significant at 12 hours. Because there is no significant mimic hypoxia at 12 hours. In the first, the induction of hypoxia was confirmed by measuring the expression level of *HIF-1a mRNA*. At the time of 12 hours, the expression of the *HIF-1a* gene was non-significantly increased compared to the control group in MOLT-4 and KG1 cell lines. Additionally, *HIF-1a* gene expression increased to approximately 5-fold after 48 hours of treatment (Figure 4). Our results indicated that the increased expression of HIF-1a contributes to the changes in the expression profiles of significant genes and miRNAs involved in cancer cell apoptosis and proliferation. According to the statistical analysis performed on the data from MOLT-4 and KG1 cells treated with cobalt chloride, during 24 and 48 hours, the results of gene expression changes after confirmation of mimic hypoxia induction by cobalt chloride were as follows:

After 24 hours mimic hypoxia induction by CoCl_2_, the expression of MRP1 and FOXM1 are significantly increased in MOLT-4 and KG1 cell lines under mimic hypoxia conditions. Additionally, the changes in the expression of BCL-xl and BBC3 were non-significant in both cell lines. The expression of the *MDR1* gene is significantly increased in the MOLT-4 cell line but the expression change of this gene was non-significant in the KG1 cell line under mimic hypoxia conditions (Figure 5).

**Figure 6:**
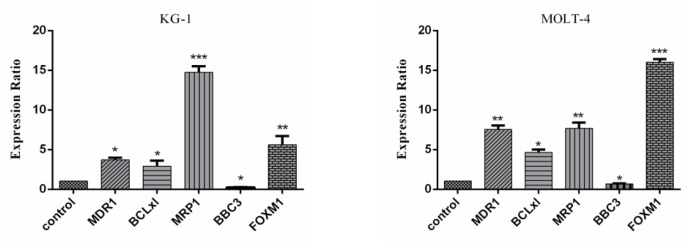
Real-Time PCR Data for MDR1, MRP1, PUMA, FOXM1, BCL-xl Expression in MOLT-4 and KG1 Cell Lines under Mimic Hypoxia Condition. The expression of these genes was evaluated by real-time PCR in MOLT-4 and KG1 cells under mimic hypoxia condition. Extraction of total RNAs were performed 48 hours after exposure to 75 and 400 μM of CoCl2 respectively. Data were obtained from the means and standard deviations of three independent experiments. *Statistically significant difference in comparison with respective data of MOLT-4 control cells (P<0.05)

At the time of 48 hours, the expression of MDR1, MRP1, FOXM1, and BCL-xl are significantly increased in MOLT-4 and KG1 cell lines under mimic hypoxia conditions. Additionally, the expression of BBC3 is decreased in both cell lines under mimic hypoxia conditions (Figure 6).

*The impact of mimic hypoxia on miRNAs expression profile in MOLT-4 and KG1 cell lines*

**Figure 7:**
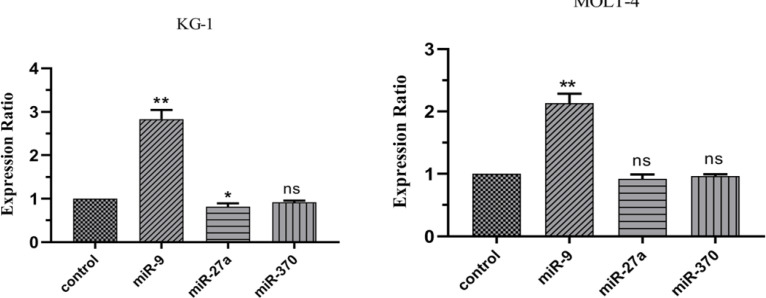
Real-Time PCR Data for miR-9, miR-370 and miR-27a Expression in MOLT-4 and KG1 Cell Lines under Mimic Hypoxia Condition. The expression of these miRNAs was evaluated by real-time PCR in MOLT-4 and KG1 cells under mimic hypoxia condition. The extraction of RNAs were performed 24 hours after exposure to 75 and 400 μM of CoCl_2_ respectively. Data were obtained from the means of three independent experiments. *Statistically significant difference in comparison with respective data of KG1 control cells (P<0.05).

In this study, the expression of three important drug resistance- and apoptosis-related miRNAs, including miR-9, miR-370 and miR-27a, were evaluated under mimic hypoxia conditions. After 24 hours, the expression of miR-9 that targets MDR1 and PUMA is significantly increased compared to the control group in MOLT-4 and KG1 cell lines under mimic hypoxia conditions. The expression changes of two other miRNAs, including miR-370, and miR-27a that target FOXM1 and MRP1, respectively, were non-significant in the MOLT-4 cell line. The expression of miR-27a is significantly decreased in the KG1 cell line (Figure 7).

**Figure 8:**
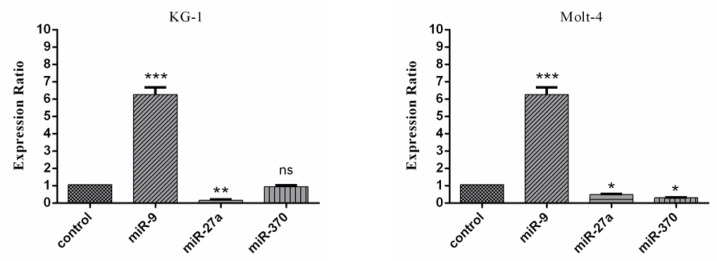
Real-Time PCR Data for miR-9, miR-370 and miR-27a Expression in MOLT-4 and KG1 Cell Lines under Mimic Hypoxia Condition. The expression of these miRNAs was evaluated by real-time PCR in MOLT-4 and KG1 cells under mimic hypoxia condition. The extraction of RNAs were performed 48 hours after exposure to 75 and 400 μM of CoCl_2_ respectively. Data were obtained from the means of three independent experiments. *Statistically significant difference in comparison with respective data of KG1 control cells (P<0.05)

Real-time PCR data also showed that after 48 hours mimic hypoxia induction, the expression of miR-9 is significantly increased in both cell lines under mimic hypoxia conditions. Additionally, the expression of miR-27a is significantly decreased. A significant decrease in the expression of miR-370 was also demonstrated in the MOLT-4 cell line but the expression of miR-370 was non-significant in the KG1 cell line (Figure 8).

**Table 1:** List of Primers for Genes and miRNAs

Gene	Primer sequence	Primer efficiency	Amplicon Size (bp)	GENE LOCUS	Annealing temperature
ATP Binding Cassette Subfamily C Member 1 (MRP1)	F: CGTGTTGGTCTCTGTGTTCCTG R: AGAAAGATGCTCTCTGGGTTTG	97.95%	181	NC_000016.10	57
ATP Binding Cassette Subfamily B Member 1 (MDR1)	F: ATTGCTCACCGCCTGTCCACC R: TGCTGATGCGTGCCATGCTCC	93%	89	NC_00000.14	57
forkhead box proteinM1 ( FOXM1)	F: TGCAGCTAGGGATGTGAATCTTC R: GGAGCCCAGTCCATCAGAACT	97.59%	188	NC_000012.12	60
Hypoxia-Inducible Factor (HIF)-1	F: GTACCCTAACTAGCCGAGGAAGAA R: GTGAATGTGGCCTGTGCAGT	98.53%	80	NC_000014.9	57
P53 Up regulated Modulator of Apoptosis (PUMA)	F: CTCTCCCTCCTCCTTCACTCT R: TCACGTTTGGCTCATTTGCTCT	98.35%	100	NC_000019	54
BCL2 Like 1 (Bcl-xL))	F: ATCCCAGCTCCACATCACC R: CGATCCGACTCACCAATACCTG	97.615	173	NC-000020.11	64
Actin beta	F: AGCATCGGGTGATGTTCATT R: ATTACAAGCATGCGTCACCA	94.51%	107	NC_000007.14	57
miR-27a	F: TTCACAGTGGCTAAG R: GTGCAGGGTCCGAGG	97.53%	78	NC_000019.10	48
miR-9	F: TAAGGCACGCGGTGAATGCC R: CTCATAGACCTACATAACGAAACA	98.68%	89	NC_000001.11	48
miR-370	F: AAGGGATCCTACTTGAGGGATGGGCGATA R: TCAAAGCTTCCCGAGCTCTGGTGTTAGAC	95.72%	75	NC_000014.9	48

## Discussion

Despite invasive treatments, many cancers become resistant to treatment. Many studies have reported defects in apoptosis and proliferation pathways in various cancer cells, contributing to the cancer cells’ resistance to treatment. This has especially been seen in leukemic cells (Manson et al., 2005). Today, hypoxia has been considered a significant factor in the growth and development of tumor cells. Most tumor cells are under hypoxia conditions. Therefore, the induction of HIF-1a leads to changes in the expression profile of genes and miRNAs. These changes in the expression of genes and miRNAs might lead to tumor suppression or progression (Benito et al., 2013; Kaur et al., 2005; Teicher, 1994). Several studies have confirmed the role of hypoxia in resistance to treatment in other cell lines, and have shown that hypoxia is associated with drug resistance and increased cell survival (Benito et al., 2013).

Our results confirmed the effect of mimic hypoxia on the expression of drug resistance-related genes. The action of CoCl_2_ differs from a decrease in pO_2_. Mechanistically, CoCl_2_ inhibits the activity of prolyl hydroxylase domain enzymes (PHDs) and contributes to the creation of a mimic hypoxia condition. Concerning to the genes involved in drug resistance, the expression of* MDR1* and *MRP1 *genes increased in both cell lines under mimic hypoxia conditions. The significant increase in the expression of *MDR1* and *MRP1* genes in the MOLT-4 and KG1 cell lines demonstrates that the mimic hypoxia plays an important role in the regulation of these genes. Considering the fundamental roles of* MDR1* and *MRP1* genes in drug resistance, hypoxia might be the cause of drug resistance in both cell lines. However, this issue needs further investigations along with a chemotherapy agent. Previous studies have shown that increased expression of FOXM1 is highly associated with drug resistance, reduces cellular sensitivity, and protects cancer cells from induced apoptosis by chemotherapy agents (Xie et al., 2015; Zhou et al., 2013).

*PUMA* and *BCL-xl* belong to the *BCL*_2_ family, two important genes that have a key role in the cancer cell apoptosis process. PUMA increases apoptosis and sensitivity of cancerous cells by binding to the BCL-xl. BCL-xl also binds to mitochondrial-free cytochrome and prevents cell apoptosis (Abedi et al., 2014; Findley et al., 1997). In this study, the evaluation of PUMA and BCL-xl expression in leukemic cell lines showed that the expression of the *PUMA* gene is significantly decreased in both cell lines. Additionally, under mimic hypoxia conditions, the expression of BCL-xl that is inhibited by PUMA is significantly increased in both cell lines. Therefore, considering the fundamental roles of PUMA and BCL-xl in apoptosis, hypoxia might affect the drug resistance of cancer cells by regulating the apoptosis related genes; however, investigations are necessary. 

The role of miRNAs, as small, non-coding RNAs in tumor cell susceptibility to chemotherapy has been reported, especially in leukemic cells (Fan et al., 2016; Han et al., 2016; Sugita et al., 2014). MiR-9, miR-370, miR-27a, three important miRNAs, belong to the HRMs, with a fundamental role in drug resistance regulation. Genes involved in cell sensitivity to the chemotherapy agents, including *PUMA, MDR1, FOXM1,* and *MRP1*, are the target genes for *miR-9*, *miR-370*, and *miR-27a*, respectively (Feng et al., 2011; Li et al., 2017; Wang et al., 2016; Zhou et al., 2013). Our results showed that, under mimic hypoxia conditions, the expression level of miR-9 is significantly increased in both cell lines, whereas the miR-9 targets, including MDR1 and PUMA, are significantly increased and decreased in both cell lines, respectively. The mechanism by which HIF-1a contributes to the changes in the expression of genes is through binding to the hypoxia-responsive elements (HREs) on the promoter of genes. Recent studies have confirmed the presence of HREs on the promoter of MDR1 and MRP1 genes. So, the possible mechanism for the alteration of gene expression in this study is the binding of HIF-1a to HRE regions. In addition, the* p53* gene also contains HREs. The *p53* gene causes overexpression of *PUMA *(Comerford et al., 2002; Cosse, Ronvaux, Ninane, Raes, and Michiels, 2009; Gottlieb and Vousden, 2010; Hammond and Giaccia, 2005; Lv et al., 2015; Sendoel and Hengartner, 2014). It is possible that HIF-1a binding to HREs in the *p53* gene promoter leads to the suppression of p53 and consequently, the reduction of *PUMA* expression. It seems the increased expression of miR-9 has also led to the suppression of PUMA. Finally, considering what was discussed above, mimic hypoxia has promoted the expression of drug resistance-related genes in both cell lines. However, further studies are needed to confirm this mechanism in altering gene expression under chemical hypoxia.

The expression level of miR-27a is significantly decreased in MOLT-4 and KG1 cell lines under mimic hypoxia conditions. Additionally, the expression of the *MRP1* as a target gene for miR-27a is increased compared to control cells in the KG1 cell line. As mentioned, the expression of the *MRP1* gene may also be increased through HIF-1a binding to the *MRP1 *gene promoter.

The expression of miR-370 is non-significantly decreased in the KG1 cell line and significantly in the MOLT-4 cell line under mimic hypoxia conditions. It seems following the suppression of miR-370 by HIF-1a, the expression of FOXM1 in both cell lines is increased. Additionally, further investigations are necessary. Since the suppression of miR-370 in hypoxic KG1 cells was non-significant, it seems the increased expression of the *FOXM1* gene in hypoxic KG1 cells is only due to the binding of HIF-1a to the FOXM1 promoter. Additionally, the non-significant change in the expression of miR-370 has not influenced the expression of FOXM1. Similarly, the expression of FOXM1 in hypoxic MOLT-4 cells was very high compared to control cells. It seems that a significant decrease in the expression of miR-370 and the binding of HIF-1a to the promoter of the *FOXM1 *gene have influenced the expression level of FOXM1 in MOLT-4 cells. Therefore, hypoxia contributes to the failure of treatment through complex pathways and the regulation of miRNAs and genes involved in apoptosis, drug resistance, or cell survival genes. However, further studies are needed to confirm this mechanism. 

Recent evidence suggests that hypoxia promotes cellular proliferation in leukemia cells. Therefore, HIF-1a plays a key role in inducing cell proliferation by regulating the expression of genes (Kaur et al., 2005; Wellmann et al., 2004). However, according to our results, mimic hypoxia reduced cell proliferation in KG1 and MOLT-4 cells, which seems to be due to the limitations of our study. Hypoxia seems to increase cell proliferation and resistance to treatment, but CoCl_2_ causes cytotoxic effects. It is also possible that, if there was not any mimic hypoxia-induced resistance, the effects of CoCl_2_ in the killing of the cells might have been much higher. Therefore, this issue needs further studies to determine the exact mechanism of mimic hypoxia induced by CoCl_2_ in the resistance of tumor cells to treatment. However, in most studies, CoCl_2_ is used to induce mimic hypoxia, while CoCl_2_ has a binding activity to DNA. In fact, the mechanism of CoCl_2_ in creating hypoxia is different. Additionally, it has extremely toxic effects and leads to fundamental changes in cell biology (Huang et al., 2003). As our results showed the toxic effects of CoCl_2_ in the concentration of 400 and 75 µM was very low for 48 hours. After 48 hours, the toxic effects of CoCl_2_ occurred; consequently, fundamental changes occur in cell biology. Accordingly, the best time to evaluate the effect of mimic hypoxia on the expression of genes involved in cellular processes is during the first 48 hours after treatment.

One of the major problems in the treatment of cancer is the failure of treatment induced by hypoxia. Therefore, targeting hypoxia is very important in cancer treatment. Target therapy using microRNAs has also been considered an important field in cancer therapy by researchers. Identification of important microRNAs in the treatment of cancer as well as diagnosis, and prognosis of treatment is very important. Hypoxia alters the expression of microRNAs involved in cancer cell development processes. Accordingly, it is very important to identify key microRNAs that change under hypoxic conditions and thus can be used in cancer therapy, diagnosis and prognosis. It is also important to look for a way to combat hypoxia and prevent it from developing in cancer cells, which will probably increase the sensitivity of the cancer cell to treatment. In addition, the hypoxia pathway may be initiated by microRNAs. Therefore, by targeting some key microRNAs, it is possible to modulate the hypoxia pathway and achieve great success in cancer treatment.

In conclusion, treatment with CoCl_2_ has an inhibitory effect on the proliferation of MOLT-4 and KG1 cell lines independent of hypoxia. This appears to be due to the toxic effect of CoCl_2_. To inhibit prolyl hydroxylase enzymes and induce the expression of HIF-1a, the cells should be treated with very low toxic concentrations of CoCl_2_. Even very low concentrations of CoCl_2_ have a somewhat toxic effect, whereas there is no such toxic effect in a real hypoxia condition. Additionally, the production of energy from the anaerobic pathways in the cancer cell under a real hypoxic condition results in increased cell proliferation. Furthermore, mimic hypoxia regulates genes and miRNAs involved in apoptosis, cell survival, and drug resistance-related genes in the MOLT-4 and KG1 cell lines. Finally, we still have a long way before we discover the exact functional mechanisms of hypoxia on gene expression, but these evaluations can provide new perspectives into this field for the upcoming studies.

## Author Contribution Statement

The authors confirm contribution to the paper as follows: study conception and design: Saeed Solali and Mohammad Reza Alivand; data collection: Behnam Emamgolizadeh; analysis and interpretation of results: Ali Mohammadi and behnam Emamgolizadeh; draft manuscript preparation: Behnam Emamgolizadeh and Ali mohammadi. All authors reviewed the results and approved the final version of the manuscript. 
